# MetStabOn—Online Platform for Metabolic Stability Predictions

**DOI:** 10.3390/ijms19041040

**Published:** 2018-03-30

**Authors:** Sabina Podlewska, Rafał Kafel

**Affiliations:** Institute of Pharmacology, Polish Academy of Sciences, Department of Medicinal Chemistry, Smętna Street 12, 31-343 Kraków, Poland; rafal.kafel@gmail.com

**Keywords:** metabolic stability, machine learning, ChEMBL database, regression, classification

## Abstract

Metabolic stability is an important parameter to be optimized during the complex process of designing new active compounds. Tuning this parameter with the simultaneous maintenance of a desired compound’s activity is not an easy task due to the extreme complexity of metabolic pathways in living organisms. In this study, the platform for *in silico* qualitative evaluation of metabolic stability, expressed as half-lifetime and clearance was developed. The platform is based on the application of machine learning methods and separate models for human, rat and mouse data were constructed. The compounds’ evaluation is qualitative and two types of experiments can be performed—regression, which is when the compound is assigned to one of the metabolic stability classes (low, medium, high) on the basis of numerical value of the predicted half-lifetime, and classification, in which the molecule is directly assessed as low, medium or high stability. The results show that the models have good predictive power, with accuracy values over 0.7 for all cases, for Sequential Minimal Optimization (SMO), k-nearest neighbor (IBk) and Random Forest algorithms. Additionally, for each of the analyzed compounds, 10 of the most similar structures from the training set (in terms of Tanimoto metric similarity) are identified and made available for download as separate files for more detailed manual inspection. The predictive power of the models was confronted with the external dataset, containing metabolic stability assessment via the GUSAR software, leading to good consistency of results for SMOreg and Naïve Bayes (~0.8 on average). The tool is available online.

## 1. Introduction

During the drug design process, attention is initially placed on obtaining the desired affinity with the appropriate receptors. However, failures of compounds at later stages of drug development are connected with other unfavorable physicochemical, pharmacokinetic, or toxic properties. The proper evaluation of these properties *in silico* is therefore just as important as the development of computational tools for accurate activity predictions [1,2,3,4].

A number of parameters can be set on the basis of which drug-like potential of the compounds is evaluated. One of the most popular groups of these properties are Lipinski’s Rule of Five, which are one of the simplest and usually the first filters applied to disqualify compounds with an unfavorable physicochemical profile [5]. It is also important to provide proper compound solubility (both in water due to solubility in the fluids in the organism, and in non-polar solvents due to the provision of penetration of biological membranes, as well as the provision of proper equilibrium between the solubility in these two environments [6]), determine the ionization potential [7] and assure that the compound can penetrate the gut-blood and blood-brain barrier (in the case of drugs acting within the central nervous system) [8]. The permeability through the biological membranes is vital not only from the point of view of determining the proper therapeutic dose but also because of the possible toxic effects. It is also important to analyze whether the compound will bind to the plasma proteins [9] as well as evaluate the half-life time or potential metabolic routes [3]. In terms of toxicity, predictions most often concern the possible interactions of the examined compound with other therapeutics and the possible undesirable modulations of other protein activities, such as hERG potassium channels [10] leading to the compound’s cardio toxicity [11,12].

These abovementioned properties are connected with the characterization of compounds in terms of their Absorption, Distribution, Metabolism, Excretion, Toxicity (ADMET) properties [3,13,14,15,16]. Metabolism and metabolic stability are particularly considered in the study, as compounds need to have sufficient time to induce the desirable therapeutic effect. Additionally, although metabolites might possess desirable biological activity, transformations of biologically active substances can also lead to the formation of toxic products. Unfortunately, the *in silico* examination of metabolic stability is very difficult due to the extreme complexity of the metabolism process. However, although it is difficult to obtain a broad predictive model that can correctly evaluate compounds that cover a wide structural spectrum, studies on metabolic stability, as well as the construction of *in silico* tools that enable the computational evaluation of metabolic stability, are continuously carried out.

A number of approaches for the prediction of ADMET properties are already available. They are mostly ligand-based tools, and two classes of models are constructed—classification ones (mutagenesic/non-mutagenesic, stable/unstable, soluble/insoluble, etc. [17,18,19]) or regression tools [19,20,21]. The application of the tools of the latter type is connected with the formation of the QSAR-type [22,23,24,25] models, in which the quantitative impact of particular structural moieties on considered parameters is examined. Many comprehensive software packages for ADMET properties evaluation are available, such as ADMET Predictor [26], CASE ULTRA [27], DEREK [28], META-PC [29], METEOR [30,31], ONCOLOGIC [32], PASS [33], TOPKAT [34], and GUSAR [35]. Moreover, the initial characteristics of physicochemical and pharmacokinetic properties are offered in most packages of software for molecular modeling, such as QikProp in the Schrödinger Suite [36], Molecular Descriptors in the MOE [37], or ADMET and Predictive Toxicology from the BIOVIA Discovery Studio [34]. A number of individual ADMET properties can also be evaluated via various online servers, such as ALOGPS [38], Molinspiration [39] PreADMET [40], MetaPrint2D [41,42,43], MetaPred [44] or Pred-hERG [45]. A summary of some of the available tools is shown in Table 1.

In this study, we focus on one of the compound’s parameters—metabolic stability. Usually, the tools for ADMET properties evaluation do not consider this property, as its predictions are very difficult due to the extreme complexity of the metabolic stability phenomenon and the great number of factors influencing this parameter. On the other hand, metabolic stability is a very important parameter, as the compound requires sufficient time to trigger the desired pharmacological response before its decomposition and moreover, the formed metabolites might not only be unable to provide the biological action of interest, but they can be toxic. For the evaluation of metabolic stability on the basis of experimental data, the most often used are half-lifetime (T_1/2_) data produced in assays using liver microsomes. There were already several machine learning-based approaches to construct general QSAR models for prediction of this parameter [46,47,48,49] and several other studies based on narrower chemical space of selected classes of compounds [50,51,52,53,54].

Here, we present a freely available online tool for metabolic stability predictions expressed as T_1/2_ or clearance. The tool uses ligand-based methodology and six machine learning methods to evaluate the compound stability, with separate models constructed for various species and assays preformed on liver microsomes and plasma. Additionally, an analysis of available metabolic stability data was performed and the most similar compounds from the training set are provided for each of the submitted structures, enabling manual analysis of the results and comparisons. The outcome of the constructed tool was compared with the external National Cancer Institute (NCI) dataset containing a GUSAR-based evaluation of metabolic stability [35]. The tool and all obtained results are freely available at http://skandal.if-pan.krakow.pl/met_stab_pred/.

## 2. Materials and Methods

The data for the construction of the tool for metabolic stability predictions were collected from the ChEMBL database version 23 [55]. All records with the T_1/2_ and intrinsic clearance parameters reported were downloaded. Data preprocessing involved the following steps: selection of *in vitro* assays performed on liver microsomes or plasma, selection of records with standard unit “hr” for T_1/2_ and “mL·min^−1^·g^−1^”, and division into separate sets referring to human, rat and mouse experiments. Due to the lack of sufficient number of entries for clearance data obtained via plasma-based assays, for plasma data, only records for T_1/2_ were gathered. The number of data points present in the respective datasets is gathered in Table 2.

The compounds were represented with the use of the 1- and 2-dimensional PaDEL-Descriptors [56] (1d2d descriptors) and Extended Fingerprint [57] (ExtFP) from the same software package. These forms of representation were chosen after initial studies performed on a series of long-chain aryl piperazines, where several fingerprints implemented in PaDEL (Extended Fingerprint, MACCS Fingerprint [58], Klekota-Roth Fingerprint [59], Graph Fingerprint [57], PubChem Fingerprint [60], and Substructure Fingerprint [61]) and descriptor sets were tested. Three dimensional descriptors were not included due to the relatively high fraction of compounds for which they could not be calculated due to errors in their generation.

The constructed tool predicts the numerical value of metabolic stability with the predictive model based on the application of the two types of machine learning algorithms, regression and classification. From the first group of methods, one algorithm was used: SMOreg, which is a modification of the very popular and efficient algorithm Support Vector Machine (SVM) [62] into Sequential Minimal Optimization (SMO) [63] and adjusted for performing regression tasks. Additionally, five classification algorithms were used: SMO, Random Forest [64], Naïve Bayes [65], k-nearest neighbor (IBk) [66], and decision tree J48 [67]. However, in order to enable easier interpretability of the outcome of regression experiments, compounds are also divided into three classes according to metabolic stability values—low, medium, and high—and the results are colored accordingly. For each of the analyzed structures, the 10 most similar compounds from the training set (Tanimoto metric [68], topological fingerprint from RDKit package [69]) are found and provided in separate files for manual inspection (the particular chemical structure is provided only once, and the median half-lifetime value is given). Structures for analysis online can be submitted as a structure data file (sdf) or drawn using the MarvinJS [70] plugin. Regardless of the way the query is submitted, all the structures are shown in the results.

## 3. Results and Discussion

### 3.1. The Importance of Separate Models for Different Species

**Figure 1:**
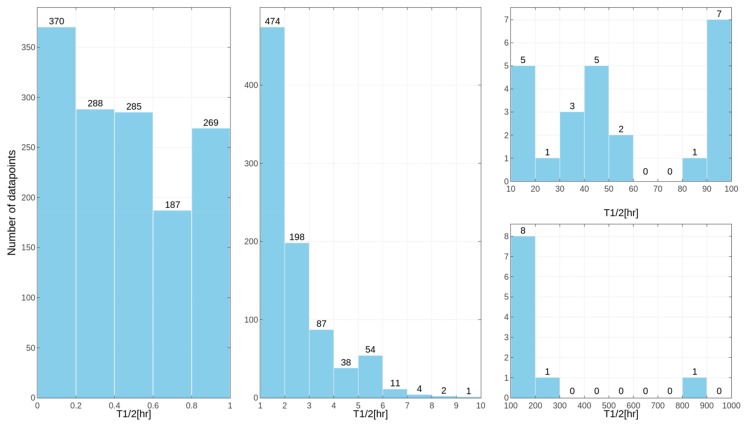
Distribution of compound half-lifetimes in the constructed datasets referring to experiments performed on human samples. For better visualization, the dataset was divided into several parts.

**Figure 2:**
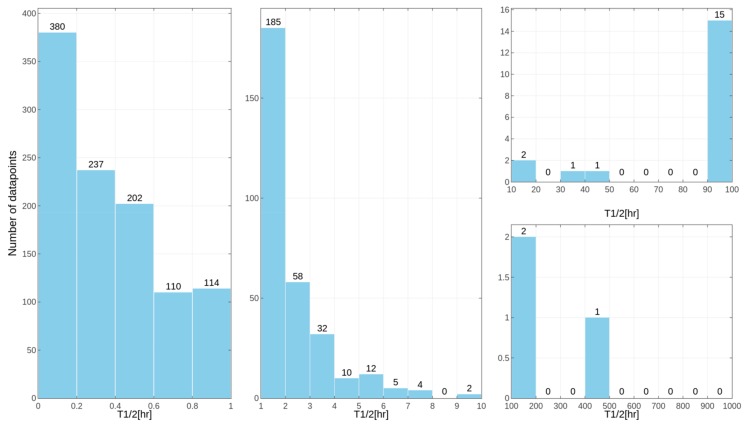
Distribution of compound half-lifetimes in the constructed datasets referring to experiments performed on rat samples. For better visualization, the dataset was divided into several parts.

**Figure 3:**
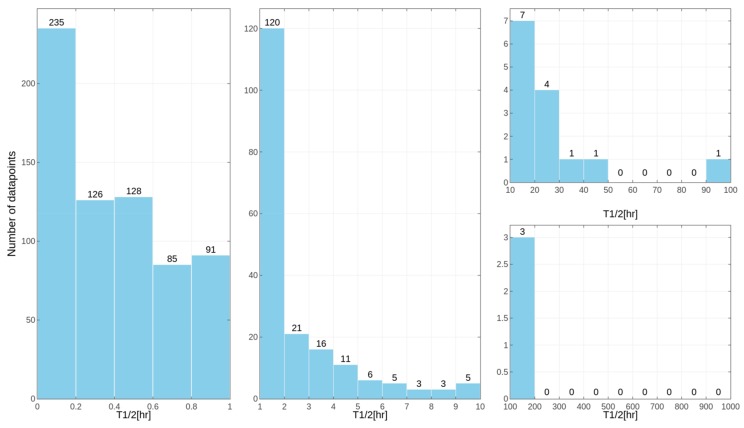
Distribution of compound half-lifetimes in the constructed datasets referring to experiments performed on mouse samples. For better visualization, the dataset was divided into several parts.

**Figure 4:**
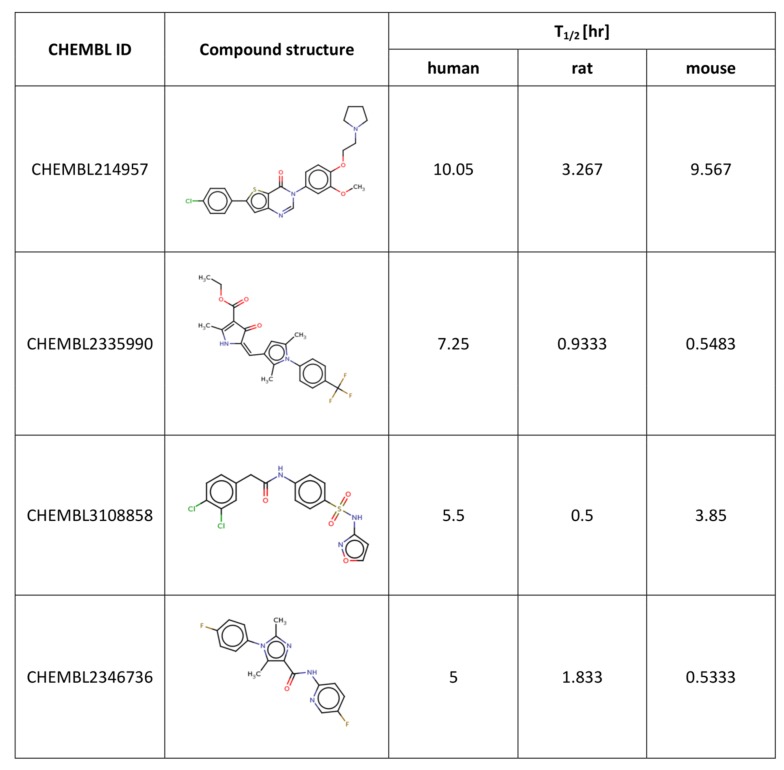
Examples of differences in results of metabolic stability tests for human, rat and mouse models.

Separate models were constructed for different species. This approach was applied due to the relatively high differences in results of the *in vitro* tests for some of the compounds, despite the similar overall distribution of data points for human, rat and mouse data with the majority of very unstable compounds (Figure 1, Figure 2 and Figure 3 presenting the distribution of T_1/2_). Some examples of the abovementioned problems are shown in Figure 4.

For all the compounds presented in the Figure 4, there were substantial differences in the results of metabolic stability examinations based on different species. For example, for compound CHEMBL214957, the human and mouse-based data were quite consistent (with stability experiments outcome being 10.05 and 9.57 hr, respectively); however, for experiments, in which rat liver microsomes were used, the obtained T_1/2_ value was equal to 3.27. For compound CHEMBL2335990, more consistency was observed for rat and mouse-based experiments with the stability values of 0.93 and 0.55 hr, respectively, whereas in experiments using human microsomes, the obtained T_1/2_ value was equal to 7.25 hr. For the other two compounds presented in Figure 4, that is CHEMBL3108858 and CHEMBL2346736, no similarities between any two experiments were observed, and the T_1/2_ values varied from 0.5 to 5.5 hr.

Therefore, the data referring to human, rat and mouse-based experiments were not mixed, and separate models were constructed for each of these experimental conditions.

In order to provide a more general picture of variations in experimental results of metabolic stability for different models (human, rat, mouse), all compounds for which the half-lifetimes were provided for all the models were identified and standard deviations (σ) given by the following equation were calculated:

(1) $$\sigma =\sqrt{\frac{\sum_{i}^{3}{\left(x_{i}-µ\right)}^{2}}{3}}$$

where *x_i_* is half-lifetime value measured for a particular model; µ—mean value of all three measures (human, mouse, rat) provided for a particular compound

**Figure 5:**
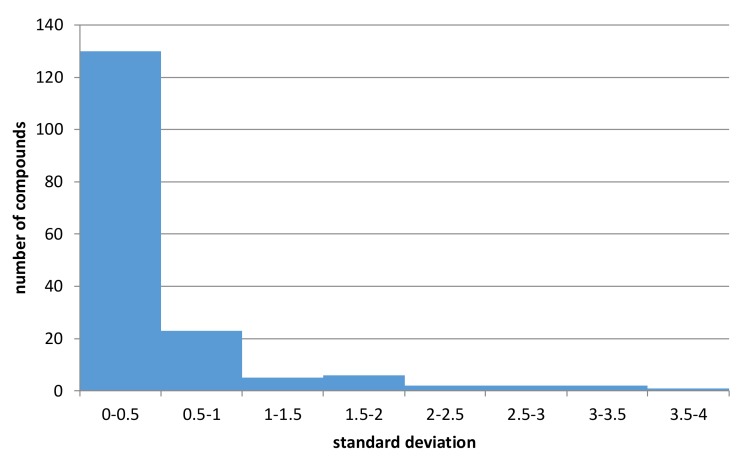
Standard deviation values of half-lifetimes between human, rat and mouse data.

The obtained values of standard deviations were presented in Figure 5 (the CHEMBL identifiers of compounds with metabolic stability data for human, rat, and mouse models with standard deviation values are provided in the Supplementary Materials).

The analysis of the histogram in Figure 5 indicates and confirms the relatively high variation of data. Out of 249 structures containing data referring to all three experimental conditions (human, rat, mouse) to which Figure 5 refers, for over 16% of them, standard deviation of half-lifetimes values was higher than 0.5, leading to swap of metabolic classes. Moreover, for 172 structures, it was above 0. Additionally, although for datasets containing human-based data, the percentage of datapoints with non-zero standard deviation was equal to 8% for mouse-based datasets, which contained much less records, the percentage of non-zero data was equal to 20%. All these results support the construction of separate predictive models for human, rat, and mouse-based data.

**Figure 6:**
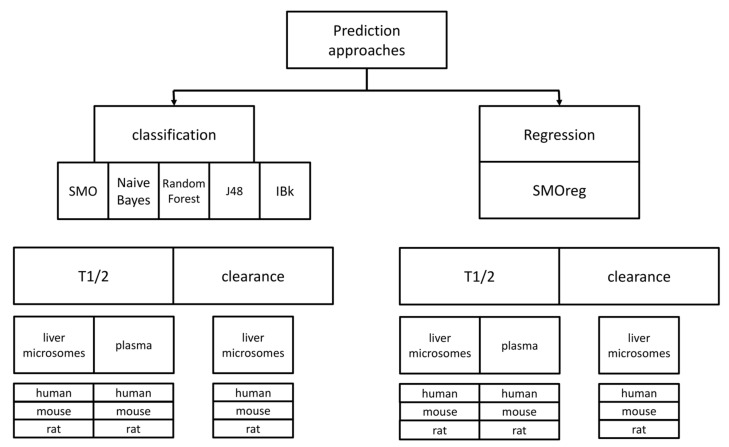
Scheme of the prediction approaches covered in the study.

A scheme of all the predictive approaches that were used in the study is presented in Figure 6. Taking into account the combination of all datasets used, prediction algorithms and compounds representation, the total number of predictive models provided as a result of the study is equal to 108; the online version of the tool for metabolic stability predictions includes 36 models referring to experimental data produced on liver microsomes and reported as T_1/2_. Remaining models are available at http://skandal.if-pan.krakow.pl/met_stab_pred/ and can directly be used within the WEKA software [71].

### 3.2. Evaluation of Models in 10-Fold Cross-Validation

Ten-fold cross-validation (CV) studies were performed for optimization and evaluation of the constructed predictive models. WEKA implementation [71] was used for all the algorithms used in the study.

The division into metabolic stability classes was applied, and evaluation parameters were subsequently calculated: overall accuracy, AUROC [72] in case of classification models, and recall and precision for each class.

The cutoffs for metabolic stability class division were as follows (T_1/2_ expressed in hours):

- ≤0.6—low
- (0.6–2.32)>—medium
- >2.32—high

The number of compounds belonging to each stability class are presented in Table 3.

The class distribution was not uniform and in all cases the highest number of compounds belonged to the group of low metabolic stability. The highest variation was observed for rat and mouse models, with 62% and 60% of compounds exhibiting low values of half-lifetimes, respectively. For both of these cases, compounds of high stability constituted 9% of the whole dataset, and the fraction of medium stability compounds in the whole dataset was equal to 29% and 31% for rat and mouse models, respectively. For human models, the compounds were more uniformly distributed among classes with 44% of compounds belonging to low and medium stability class, and the remaining 12% of datapoints referred to compounds characterized by high metabolic stability.

The values of evaluating parameters obtained in 10-fold CV studies are presented in Table 4 for T_1/2_ liver microsomes data, all results for the remaining datasets are present in the Supplementary Materials.

**Figure 7:**
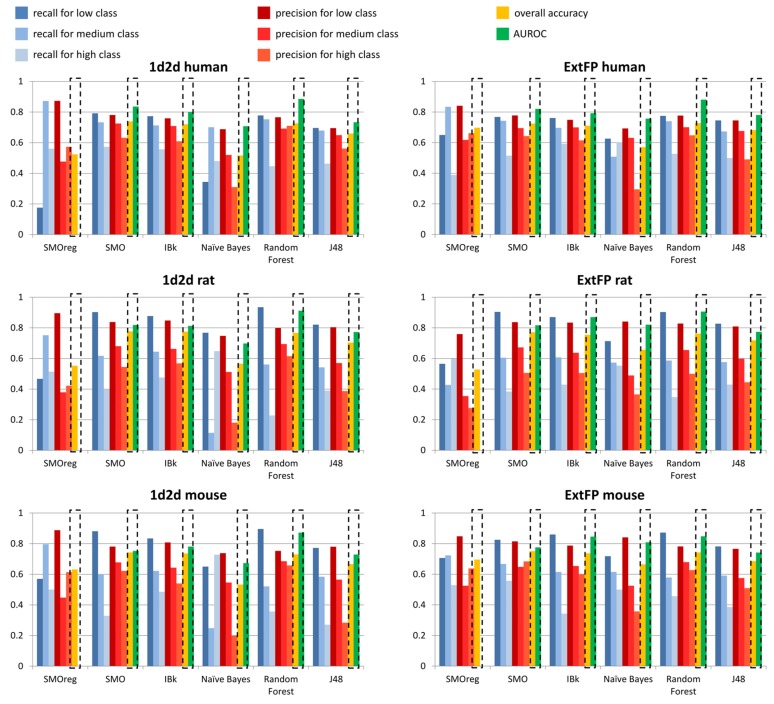
Visualization of evaluating parameters values obtained in 10-fold CV studies (T_1/2_ liver microsomes data).

The parameters values above 0.7 are presented in bold. In order to facilitate the results interpretation, the data were also presented in the respective figures (Figure 7 for T_1/2_ liver microsomes; remaining data in Supplementary Materials).

In general, the values of evaluating parameters are high and show that the constructed models are capable of making a valid evaluation of metabolic stability expressed as T_1/2_.

The general observation is that 1d2d descriptors provided higher values of evaluating parameters than ExtFP. It is also visible that there are high variations for recall and precision values depending on the metabolic stability class considered. However, for the great majority of cases, low stability class was the one that led to the highest recall and precision values. However, the highest number of cases belonged to the low stability class in all human, mouse and rat datasets. Relatively, the highest recall values were obtained for rat models, for Random Forest and SMO models (0.935 and 0.903, respectively) with compounds represented by 1d2d descriptors; for ExtFP representation, the recall values were on similar level for these methods: 0.903, and 0.904, respectively. On the other hand, it was IBk (for 1d2d descriptors) and Naïve Bayes (for ExtFP) that provided the highest precision values: 0.848 and 0.841, respectively.

Taking into account the overall accuracy of predictions, the SMO, IBk and Random Forest methods were the only ones that consistently provided overall accuracy over 0.7. For human models, it was 0.739 for SMO and 0.728 for Random Forest (for 1d2d descriptors and ExtFP representations, respectively) that were the highest overall accuracies; however, for mouse and rat data, it was SMO that provided the best performance of predictive models for both compounds representations, with values varying from 0.743 to 0.777. The other parameter that provided information on the general performance of the model was AUROC, which in general adopted higher values than overall accuracy, reaching values close to 0.9: 0.886 and 0.881 for Random Forest models constructed on human data, 0.912 and 0.906 for Random Forest models that used mouse data, and 0.872 and 0.848 for Random Forest model built on rat data (for 1d2d descriptors and ExtFP representations, respectively).

### 3.3. Comparison of the Constructed Tool Outcome with the Predictions on the External Dataset

The outcome of the constructed tool was compared with the external NCI dataset containing GUSAR-based evaluation of metabolic stability [35]. The predictions provided there were only binary (stable/unstable). After the removal of compounds with errors, and those containing heavy atoms, such as Pb, Ag, Se, Te (leading to errors in descriptors calculations), the predictions with models produced on T_1/2_ liver microsomes data were carried out (as the dataset originally contained such data). The results obtained via these two approaches were compared by accuracy indicating the fraction of the same predictions (Table 5).

The comparison of the predictions obtained via the constructed tool with the GUSAR predictions indicate great dependence of the results on the machine learning algorithm applied. The most consistent predictions with the output of the GUSAR software were provided by the SMOreg and Naïve Bayes algorithms. As the GUSAR predictions were binary (the compounds were evaluated only as stable or unstable) and our tool evaluates compounds as low, medium or high stability, two approaches of dealing with records assigned to medium stability class were applied: such entries were removed before the accuracy calculation, or the medium class assignments were shifted to the high stability class, as being more populated. With the removal of records assigned to medium stability class, the most consistent predictions with the GUSAR software outcome were obtained with the use of the SMOreg algorithm with 1d2d descriptors used for compounds representation (accuracy of 0.89). Surprisingly, the application of SMOreg with ExtFP for compounds representation led to high inconsistency of the constructed tool outcome with the GUSAR-based evaluation with the accuracy of 0.39. The GUSAR software output was also in line with the Naïve Bayes predictions, for both 1d2d descriptors and ExtFP compounds representation, with accuracies of 0.81 and 0.72, respectively. In general, for sets with medium class predictions removed, the application of 1d2d descriptors for compounds representation led to more consistent results with the GUSAR software than ExtFP (for all algorithms but IBk, the accuracies were much higher for the former compounds representation, by from 0.13 for J48, through 0.38 for Random Forest, to 0.50 for SMOreg). The advantage of the 1d2d descriptors representation over ExtFP was not visible for the situation, when the medium class predictions were manually shifted to the group of records referring to high stability compounds. For the most consistent with the GUSAR predictions, SMOreg, the difference in accuracy between these two forms of representations was only 0.01 (0.78 vs. 0.77). For Naïve Bayes, which also produced results consistent with the GUSAR software, the difference was equal to 0.05 (accuracy of 0.77 vs. 0.72 for 1d2d descriptors and ExtFP, respectively). Random Forest predictions were in line with the output of the GUSAR software only for the 1d2d descriptors (0.71 accuracy for this representation vs. 0.54 for ExtFP).

### 3.4. Example Results

**Figure 8:**
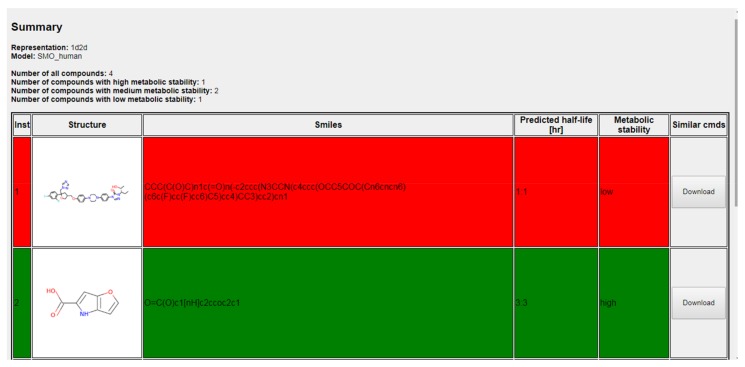
Screenshot from example results, containing summary of predictions and predicted metabolic stabilities with coloring corresponding to metabolic stability results.

A screenshot from the example output of the tool is presented in Figure 8. The summary describes the compound representation used, the predictive model applied, the number of input compounds and the number of compounds assigned to a particular metabolic stability class. Detailed results are gathered in a table with the structure and simplified molecular-input line-entry system (SMILES) of a compound, the predicted value of half-lifetime and the metabolic stability class to which a compound was assigned. Additionally, in order to perform a more detailed analysis, the 10 most similar compounds from the training set (in terms of Tanimoto metric-based similarity) can be downloaded for each of the analyzed structures (due to the computational resources limitations, this option is available for a maximum number of 100 structures submitted at one time). As the training sets contained all available data, in order to prevent situations in which the number of structures for manual inspection is very restricted, the particular chemical structure was listed only once, and in case of multiple metabolic stability entries available, median values of half-lifetimes were provided. The abovementioned data can be downloaded separately for each of the analyzed compounds or as one zipped directory.

## 4. Materials and Methods

The models’ parameters were optimized with the set of values gathered in Table 6. For each model, the value that provided the lowest overall accuracy was selected. The conditions selected for each model are shown in Table 7.

The overall accuracy, recall and precision were calculated using the following equations:

(2) $$\text{overall accuracy}=\frac{\text{number of correct predictions}}{\text{number of all predictions}}$$

(3) $$\mathrm{recall}=\frac{\mathrm{TP}}{\mathrm{TP}+\mathrm{FN}}$$

(4) $$\mathrm{precision}=\frac{\mathrm{TP}}{\mathrm{TP}+\mathrm{FP}}$$

where TP is the number of instances correctly assigned to a particular class, FN is the number of instances belonging to particular class incorrectly assigned to another one, and FP is the number of instances incorrectly assigned to particular class.

## 5. Conclusions

In summary, a tool for the qualitative evaluation of metabolic stability expressed as half-lifetime was constructed. It uses regression and classification tools to provide the assignment of a compound to a particular stability class (low, medium, high), 1d2d descriptors and ExtFP for compound representation, and the SMOreg, Random Forest, SMO, IBk, Naïve Bayes and J48 machine learning algorithms for making predictions. The tool is freely available online and allows for the submission of structures via sdf files or through drawing. Separate predictive models were constructed for human, rat and mouse data, and for data obtained in experiments using liver microsomes and plasma, as well as for data with metabolic stability expressed as T_1/2_ and clearance. A detailed retrospective analysis and the application of the constructed model to the external dataset proved the usefulness of the developed tool. The tool can be very useful in designing new potential drugs, and in enabling a fast initial evaluation of a compound’s metabolic stability.

## Figures and Tables

**Table 1:** A summary of some of the available tools for ADMET properties predictions.

Package Name	Link	Availability	Description
ADMET Predictor	http://www.simulations-plus.com/	commercial software	Comprehensive characteristic of physicochemical and ADMET properties of compounds, including cancerogenity, mutagenicity, overall toxicity and possibility of interactions with 5 selected CYP isoforms
CASE ULTRA	http://www.multicase.com/case-ultra-models	commercial software	A set of statistical and expert tools for evaluation of compounds toxicity
DEREK	http://www.lhasalimited.org	commercial software	Expert system for predicting toxicity of compounds, including cancerogenity, mutagenicity, genotoxicity, teratogenicity, influence on fertility, irritating influence on skin or allergic effect
META-PC	http://www.multicase.com/meta-pc	commercial software	Expert system for predicting products of compounds metabolism
METEOR	http://www.lhasalimited.org	commercial software	Expert system for predicting metabolic transformations
ONCOLOGIC	http://www2.epa.gov/tsca-screening-tools/oncologictm-computer-system-evaluate-carcinogenic-potential-chemicals	free software	Predicting of cancerogenicity of compounds
PASS	http://www.pharmaexpert.ru	commercial software (simplified version is freely available online)	Qualitative evaluation of above 3500 properties, including mechanisms of action, side and toxic effects, interaction with various enzymes and transport proteins, influence on genes expression
TOPKAT	http://accelrys.com/products/collaborative-science/biovia-discovery-studio/qsar-admet-and-predictive-toxicology.html	commercial software	Mutagenicity, cancerogenity, irritating action on skin, eyes, etc.
GUSAR	http://www.way2drug.com/gusar/index.html	commercial software	Evaluation of compounds toxicity and interaction with selected off-targets

**Table 2:** The number of compounds present in each dataset used for the predictive model’s construction.

	Human		Rat		Mouse	
	Liver Microsomes	Plasma	Liver Microsomes	Plasma	Liver Microsomes	Plasma
T_1/2_	2127	561	1308	277	808	62
clearance	2546	-	1244	-	266	-

**Table 3:** Statistics of number of compounds belonging to each class.

Class/Number of Compounds	Human	Rat	Mouse
Low	928 (44%)	814 (62%)	486 (60%)
Medium	937 (44%)	382 (29%)	252 (31%)
High	262 (12%)	112 (9%)	70 (9%)
Total	2127	1308	808

**Table 4:** Evaluation parameters obtained in 10-fold CV for data (T_1/2_) produced on liver microsomes. Values above 0.7 are depicted in bold.

			1d2d Descriptors						ExtFP					
		Class	SMOreg	SMO	IBk	Naïve Bayes	Random Forest	J48	SMOreg	SMO	IBk	Naïve Bayes	Random Forest	J48
human	Recall	Low	0.176	**0.792**	**0.773**	0.344	**0.778**	0.696	0.650	**0.768**	**0.761**	0.626	**0.775**	**0.745**
		Medium	**0.872**	**0.733**	**0.713**	**0.701**	**0.753**	0.679	**0.834**	**0.743**	0.697	0.508	**0.740**	0.673
		High	0.561	0.573	0.557	0.480	0.447	0.463	0.389	0.515	0.592	0.603	0.527	0.500
	Precision	Low	**0.873**	**0.781**	**0.759**	0.688	**0.766**	0.695	**0.841**	**0.778**	**0.749**	0.693	**0.777**	**0.745**
		Medium	0.477	**0.725**	**0.709**	0.520	0.692	0.649	0.618	0.695	**0.700**	0.631	**0.701**	0.676
		High	0.573	0.632	0.609	0.310	**0.710**	0.562	0.662	0.643	0.615	0.295	0.648	0.491
	Overall accuracy		0.524	**0.739**	**0.720**	0.517	**0.726**	0.660	0.698	**0.725**	**0.711**	0.571	**0.728**	0.682
	AUROC			**0.836**	**0.800**	**0.708**	**0.886**	**0.7333**		**0.821**	**0.792**	**0.757**	**0.881**	**0.781**
rat	Recall	Low	0.467	**0.903**	**0.877**	**0.768**	**0.935**	**0.821**	0.565	**0.904**	**0.870**	**0.713**	**0.903**	**0.827**
		Medium	**0.752**	0.617	0.644	0.114	0.561	0.542	0.427	0.605	0.607	0.573	0.586	0.576
		High	0.514	0.400	0.476	0.648	0.228	0.390	0.598	0.384	0.429	0.553	0.348	0.429
	Precision	Low	**0.896**	**0.838**	**0.848**	**0.748**	**0.799**	**0.804**	**0.759**	**0.837**	**0.834**	**0.841**	**0.828**	**0.809**
		Medium	0.379	0.680	0.663	0.512	0.694	0.570	0.354	0.672	0.637	0.489	0.655	0.598
		High	0.422	0.545	0.568	0.181	0.615	0.387	0.276	0.506	0.505	0.365	0.500	0.444
	Overall accuracy		0.553	**0.777**	**0.775**	0.566	**0.767**	**0.704**	0.528	**0.771**	**0.754**	0.657	**0.762**	**0.718**
	AUROC			**0.819**	**0.813**	0.698	**0.912**	**0.773**		**0.817**	**0.870**	**0.821**	**0.906**	**0.774**
mouse	Recall	Low	0.570	**0.881**	**0.834**	0.650	**0.896**	**0.772**	**0.706**	**0.825**	**0.860**	**0.718**	**0.872**	**0.782**
		Medium	**0.796**	0.601	0.622	0.248	0.521	0.584	0.723	0.667	0.615	0.615	0.579	0.591
		High	0.500	0.329	0.486	0.728	0.357	0.271	0.529	0.557	0.343	0.500	0.457	0.386
	Precision	Low	**0.888**	**0.781**	**0.808**	**0.738**	**0.753**	**0.780**	**0.848**	**0.815**	**0.787**	**0.841**	**0.782**	**0.766**
		Medium	0.448	0.678	0.643	0.546	0.685	0.565	0.525	0.648	0.654	0.525	0.679	0.575
		High	0.614	0.622	0.540	0.199	0.658	0.284	0.638	0.684	0.600	0.357	0.627	0.509
	Overall accuracy		0.632	**0.743**	**0.736**	0.533	**0.730**	0.667	0.696	**0.751**	**0.737**	0.665	**0.743**	0.686
	AUROC			**0.753**	**0.781**	0.673	**0.872**	**0.729**		**0.776**	**0.846**	**0.809**	**0.848**	**0.742**

**Table 5:** Accuracies of predictions on external test set (T_1/2_ human data on liver microsomes). Values above 0.7 are depicted in bold.

		SMOreg	SMO	IBk	Naïve Bayes	Random Forest	J48
Medium class predictions removed	1d2d descriptors	**0.89**	0.58	0.22	**0.81**	0.61	0.51
	ExtFP	0.39	0.27	0.44	**0.72**	0.23	0.38
Medium class preditcions shifted to high class	1d2d descriptors	**0.78**	0.66	0.22	**0.77**	**0.71**	0.58
	ExtFP	**0.77**	0.64	0.61	**0.72**	0.54	0.53

**Table 6:** Optimization conditions for SMOreg, SMO, Random Forest, and IBk.

Method	Parameter	Tested Values
SMOreg/SMO	C	0.01, 0.1, 1, 10, 100, 1000
	Gamma	0.001, 0.01, 0.1, 1, 10
	Operations on data	Normalization standardization
Random Forest	Number of trees	10, 100, 1000
Ibk	Number of nearest neighbors	1, 2, 3, 4, 5

**Table 7:** Conditions selected for each model.

Compounds Representation	Method	Parameter	Human	Rat	Mouse
1d2d descriptors	SMOreg	C	0.01	0.1	0.1
		gamma	0.1	0.1	0.1
		Operations on data	normalization	normalization	normalization
ExtFP		C	0.1	1	1
		gamma	0.001	0.001	0.001
		Operations on data	standardization	standardization	standardization
1d2d descriptors	SMO	C	100	100	100
		gamma	0.01	0.1	0.1
		Operations on data	normalization	normalization	normalization
ExtFP		C	10	10	10
		gamma	0.01	0.01	0.001
		Operations on data	normalization	normalization	normalization
1d2d descriptors	Random Forest	Number of trees	1000	1000	1000
ExtFP			1000	100	100
1d2d descriptors	IBk	Number of nearest neighbors	1	1	1
ExtFP			1	5	1

## Supplementary Materials

Supplementary materials can be found at http://www.mdpi.com/1422-0067/19/4/1040/s1.

## Abbreviations

1d2d	1-and 2-dimensional descriptors
ExtFP	Extended Fingerprint
SVM	Support Vector Machine
SMO	Sequential Minimal Optimization
CV	Cross-validation
