# Supplementary material for: MetStabOn—Online Platform for Metabolic Stability Predictions
Source: Int J Mol Sci. 2018 Mar 30;19(4):1040. doi: 10.3390/ijms19041040 (PMC5979396; doi:10.3390/ijms19041040)
Supplement: Supplementary file 1 [file ijms-19-01040-s001.zip › Supplementary_Material/File_S11_Text_File.docx]

|  |  |  | 1d2d descriptors | | | | | | ExtFP | | | | | |
| --- | --- | --- | --- | --- | --- | --- | --- | --- | --- | --- | --- | --- | --- | --- |
|  |  |  | **SMOreg** | **SMO** | **IBk** | **Naïve Bayes** | **Random Forest** | **J48** | **SMOreg** | **SMO** | **IBk** | **Naïve Bayes** | **Random Forest** | **J48** |
| human | **Recall** | **Low** | 0.480 | **0.748** | **0.757** | 0.128 | **0.775** | 0.670 | 0.472 | **0.728** | **0.746** | 0.478 | **0.724** | 0.688 |
|  |  | **Medium** | 0.452 | 0.486 | 0.623 | 0.290 | 0.456 | 0.478 | 0.475 | 0.465 | 0.514 | 0.576 | 0.431 | 0.375 |
|  |  | **High** | **0.827** | **0.721** | 0.642 | **0.877** | **0.749** | 0.637 | **0.857** | **0.757** | **0.704** | 0.667 | **0.746** | 0.646 |
|  | **Precision** | **Low** | **0.797** | **0.718** | **0.760** | **0.700** | **0.713** | 0.682 | **0.872** | **0.731** | **0.742** | **0.727** | **0.717** | 0.654 |
|  |  | **Medium** | 0.359 | 0.493 | 0.524 | 0.426 | 0.563 | 0.475 | 0.421 | 0.492 | 0.487 | 0.413 | 0.477 | 0.400 |
|  |  | **High** | 0.638 | **0.750** | **0.747** | 0.392 | **0.720** | 0.626 | 0.585 | **0.722** | **0.739** | 0.600 | **0.701** | 0.656 |
|  | **Overall accuracy** |  | 0.589 | 0.669 | 0.682 | 0.419 | 0.682 | 0.607 | 0.602 | 0.668 | 0.670 | 0.565 | 0.654 | 0.591 |
|  | **AUROC** |  |  | **0.807** | **0.781** | **0.759** | **0.875** | **0.728** |  | **0.803** | **0.803** | **0.766** | **0.867** | **0.764** |
| rat | **Recall** | **Low** | 0.432 | **0.722** | 0.625 | 0.458 | 0.694 | 0.556 | 0.395 | 0.631 | 0.690 | 0.452 | 0.667 | 0.548 |
|  |  | **Medium** | 0.478 | 0.623 | **0.710** | 0.438 | 0.681 | 0.609 | 0.550 | 0.634 | 0.573 | **0.707** | 0.561 | 0.512 |
|  |  | **High** | **0.798** | **0.716** | **0.739** | **0.750** | **0.761** | **0.704** | **0.802** | **0.703** | 0.631 | 0.531 | 0.685 | 0.613 |
|  | **Precision** | **Low** | 0.640 | 0.658 | **0.726** | 0.589 | **0.758** | 0.625 | **0.739** | 0.688 | 0.617 | 0.633 | 0.636 | 0.561 |
|  |  | **Medium** | 0.500 | 0.642 | 0.613 | 0.491 | 0.644 | 0.560 | 0.483 | 0.598 | 0.546 | 0.414 | 0.590 | 0.472 |
|  |  | **High** | 0.612 | **0.759** | **0.747** | 0.589 | **0.744** | 0.689 | 0.636 | 0.690 | **0.722** | **0.766** | 0.685 | 0.642 |
|  | **Overall accuracy** |  | 0.587 | 0.684 | 0.688 | 0.558 | **0.710** | 0.623 | 0.603 | 0.656 | 0.627 | 0.556 | 0.638 | 0.559 |
|  | **AUROC** |  |  | **0.818** | **0.736** | **0.785** | **0.861** | **0.720** |  | **0.911** | **0.901** | **0.880** | **0.940** | **0.888** |
| mouse | **Recall** | **Low** | 0.111 | 0 | 0.333 | 0.111 | 0 | 0.333 | 0.100 | 0 | 0.200 | 0.100 | 0 | 0.200 |
|  |  | **Medium** | 0.333 | 0 | 0.533 | 0.600 | 0.533 | 0.333 | 0.313 | 0.375 | 0.625 | 0.563 | 0.563 | 0.375 |
|  |  | **High** | **0.913** | **1.0** | 0.696 | **0.870** | **0.870** | 0.478 | **0.944** | **1.0** | **0.861** | **0.861** | **1.0** | **0.722** |
|  | **Precision** | **Low** | 0.333 | 0 | 0.300 | 0.333 | 0 | 0.231 | 0.500 | 0 | 0.33 | 0.250 | 0 | 0.286 |
|  |  | **Medium** | 0.556 | 0 | 0.571 | 0.643 | 0.615 | 0.385 | **0.833** | **1.0** | 0.667 | 0.643 | **0.818** | 0.429 |
|  |  | **High** | 0.600 | 0.489 | 0.696 | 0.667 | 0.606 | 0.524 | 0.630 | 0.643 | **0.756** | **0.705** | **0.720** | 0.634 |
|  | **Overall accuracy** |  | 0.574 | 0.469 | 0.551 | 0.612 | 0.571 | 0.388 | 0.645 | 0.656 | 0.672 | 0.641 | **0.703** | 0.531 |
|  | **AUROC** |  |  | 0.500 | 0.565 | 0.449 | 0.648 | 0.546 |  | 0.5 | 0.582 | **0.747** | **0.813** | 0.505 |

**Table 2.** Evaluation parameters obtained in 10-fold CV for data (T_1/2_) produced on plasma. Values above 0.7 are depicted in bold.
