# Supplementary material for: MetStabOn—Online Platform for Metabolic Stability Predictions
Source: Int J Mol Sci. 2018 Mar 30;19(4):1040. doi: 10.3390/ijms19041040 (PMC5979396; doi:10.3390/ijms19041040)
Supplement: Supplementary file 1 [file ijms-19-01040-s001.zip › Supplementary_Material/File_S12_Text_File.docx]

|  |  |  | 1d2d descriptors | | | | | | ExtFP | | | | | |
| --- | --- | --- | --- | --- | --- | --- | --- | --- | --- | --- | --- | --- | --- | --- |
|  |  |  | **SMOreg** | **SMO** | **IBk** | **Naïve Bayes** | **Random Forest** | **J48** | **SMOreg** | **SMO** | **IBk** | **Naïve Bayes** | **Random Forest** | **J48** |
| human | **Recall** | **Low** | 0.345 | **0.796** | **0.806** | 0.582 | **0.797** | **0.770** | 0.045 | **0.760** | **0.810** | 0.636 | **0.774** | 0.697 |
|  |  | **Medium** | 0.007 | 0.054 | 0.107 | 0.589 | 0.095 | 0.113 | 0 | 0.047 | 0.106 | 0.212 | 0.106 | 0.094 |
|  |  | **High** | **0.743** | **0.862** | **0.794** | 0.073 | **0.856** | **0.758** | **0.981** | **0.864** | **0.782** | 0.599 | **0.824** | **0.777** |
|  | **Precision** | **Low** | 0.492 | **0.777** | **0.722** | 0.538 | **0.780** | 0.693 | 0.657 | **0.762** | **0.712** | 0.586 | **0.754** | 0.689 |
|  |  | **Medium** | 0.091 | 0.474 | 0.247 | 0.077 | 0.356 | 0.123 | 0 | 0.615 | 0.243 | 0.117 | 0.198 | 0.152 |
|  |  | **High** | 0.558 | **0.788** | **0.809** | **0.835** | **0.795** | **0.762** | 0.540 | **0.773** | **0.809** | **0.719** | **0.793** | **0.748** |
|  | **Overall accuracy** |  | 0.537 | **0.780** | **0.752** | 0.314 | **0.780** | 0.693 | 0.538 | **0.767** | **0.747** | 0.588 | **0.755** | 0.699 |
|  | **AUROC** |  |  | **0.828** | **0.863** | 0.672 | **0.899** | **0.753** |  | **0.802** | **0.854** | **0.733** | **0.882** | **0.750** |
| rat | **Recall** | **Low** | 0.521 | **0.776** | **0.793** | 0.385 | **0.710** | 0.664 | 0.475 | **0.727** | **0.804** | 0.640 | **0.743** | 0.691 |
|  |  | **Medium** | 0 | 0.095 | 0.131 | 0.071 | 0.059 | 0.155 | 0 | 0.083 | 0.095 | 0.286 | 0.119 | 0.167 |
|  |  | **High** | 0.670 | **0.909** | **0.838** | **0.827** | **0.904** | **0.772** | 0.599 | **0.929** | **0.838** | 0.665 | **0.887** | **0.814** |
|  | **Precision** | **Low** | 0.475 | **0.786** | **0.706** | 0.616 | **0.764** | 0.659 | 0.407 | **0.803** | **0.718** | 0.624 | **0.764** | **0.706** |
|  |  | **Medium** | 0 | 0.348 | 0.314 | 0.069 | 0.313 | 0.137 | 0 | **0.778** | 0.250 | 0.126 | 0.256 | 0.187 |
|  |  | **High** | 0.656 | **0.832** | **0.843** | 0.674 | **0.793** | **0.787** | 0.612 | **0.798** | **0.839** | **0.795** | **0.821** | **0.794** |
|  | **Overall accuracy** |  | 0.580 | **0.805** | **0.772** | 0.617 | **0.776** | 0.690 | 0.523 | **0.799** | **0.774** | 0.629 | **0.783** | **0.726** |
|  | **AUROC** |  |  | **0.853** | **0.873** | **0.710** | **0.888** | **0.739** |  | **0.816** | **0.879** | **0.776** | **0.891** | **0.771** |
| mouse | **Recall** | **Low** | 0 | 0.677 | 0.645 | 0.597 | 0.532 | 0.613 | 0 | 0.597 | 0.567 | 0.597 | 0.627 | 0.552 |
|  |  | **Medium** | 0 | 0.400 | 0.400 | 0.467 | 0.267 | 0.333 | 0 | 0.400 | 0.400 | 0.400 | 0.400 | 0.400 |
|  |  | **High** | **1.0** | **0.934** | **0.889** | **0.724** | **0.950** | **0.829** | **1.0** | **0.984** | **0.929** | **0.886** | **0.940** | **0.875** |
|  | **Precision** | **Low** | 0 | **0.764** | 0.635 | 0.552 | **0.767** | 0.559 | 0 | **0.930** | **0.745** | 0.689 | **0.808** | 0.673 |
|  |  | **Medium** | 0 | 0.667 | 0.667 | 0.194 | 0.571 | 0.333 | 0 | **0.857** | 0.600 | 0.461 | 0.545 | 0.333 |
|  |  | **High** | 0.577 | **0.871** | **0.866** | **0.845** | **0.827** | **0.857** | 0.692 | **0.838** | **0.834** | **0.836** | **0.852** | **0.834** |
|  | **Overall accuracy** |  | 0.577 | **0.835** | **0.796** | 0.673 | **0.804** | **0.742** | 0.692 | **0.847** | **0.802** | **0.780** | **0.825** | **0.761** |
|  | **AUROC** |  |  | **0.781** | **0.809** | **0.716** | **0.812** | **0.704** |  | **0.795** | **0.771** | **0.784** | **0.840** | 0.695 |

**Table 3**. Evaluation parameters obtained in 10-fold CV for data (clearance) produced on liver microsomes. Values above 0.7 are depicted in bold.
