# Supplementary figures and images for: MetStabOn—Online Platform for Metabolic Stability Predictions
Source: Int J Mol Sci. 2018 Mar 30;19(4):1040. doi: 10.3390/ijms19041040 (PMC5979396; doi:10.3390/ijms19041040)

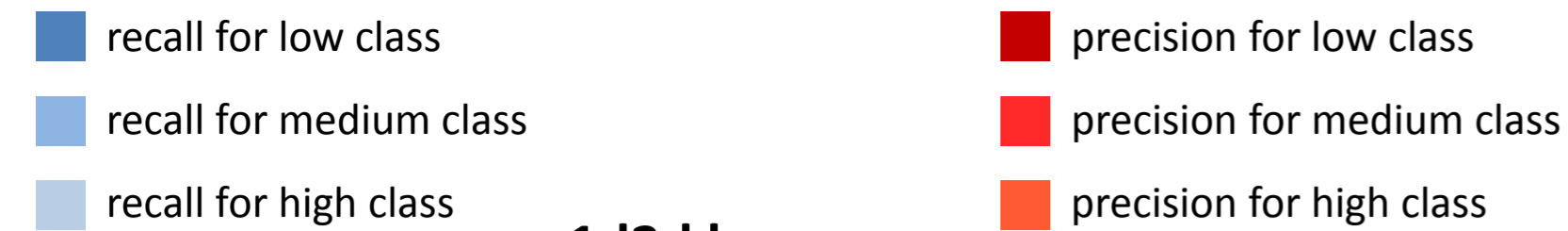

**1d2d human**

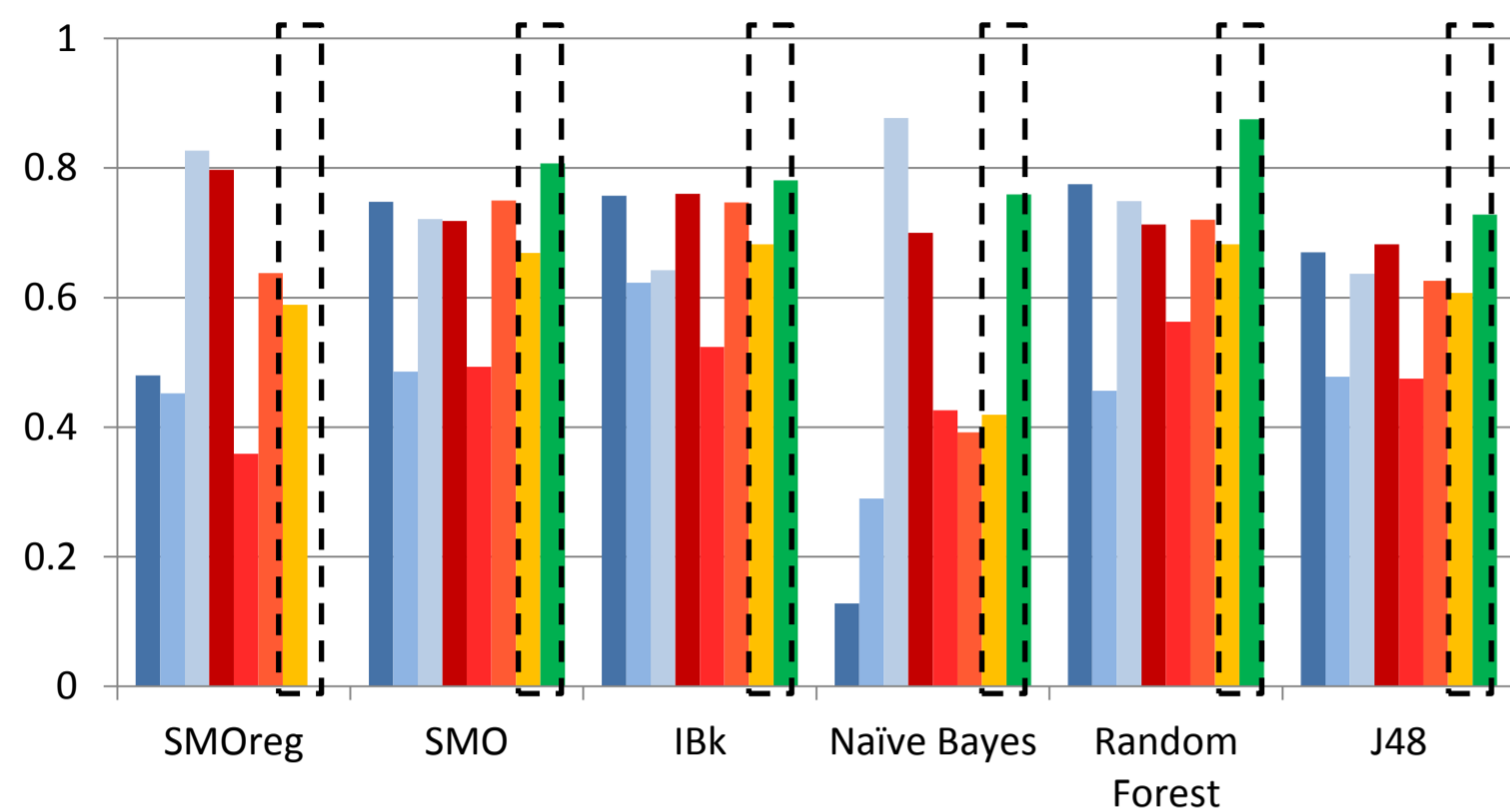

**ExtFP human**

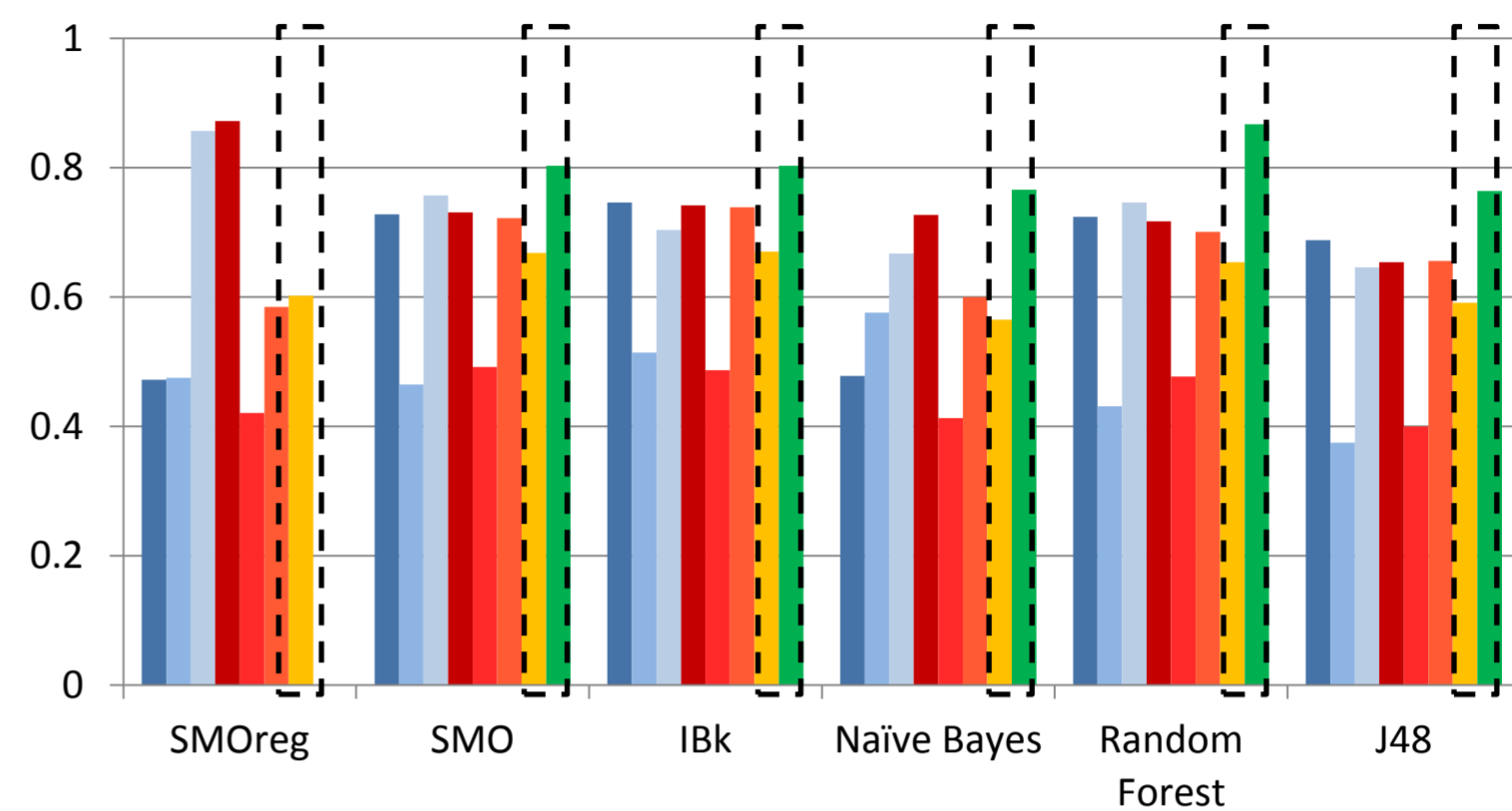

**1d2d rat**

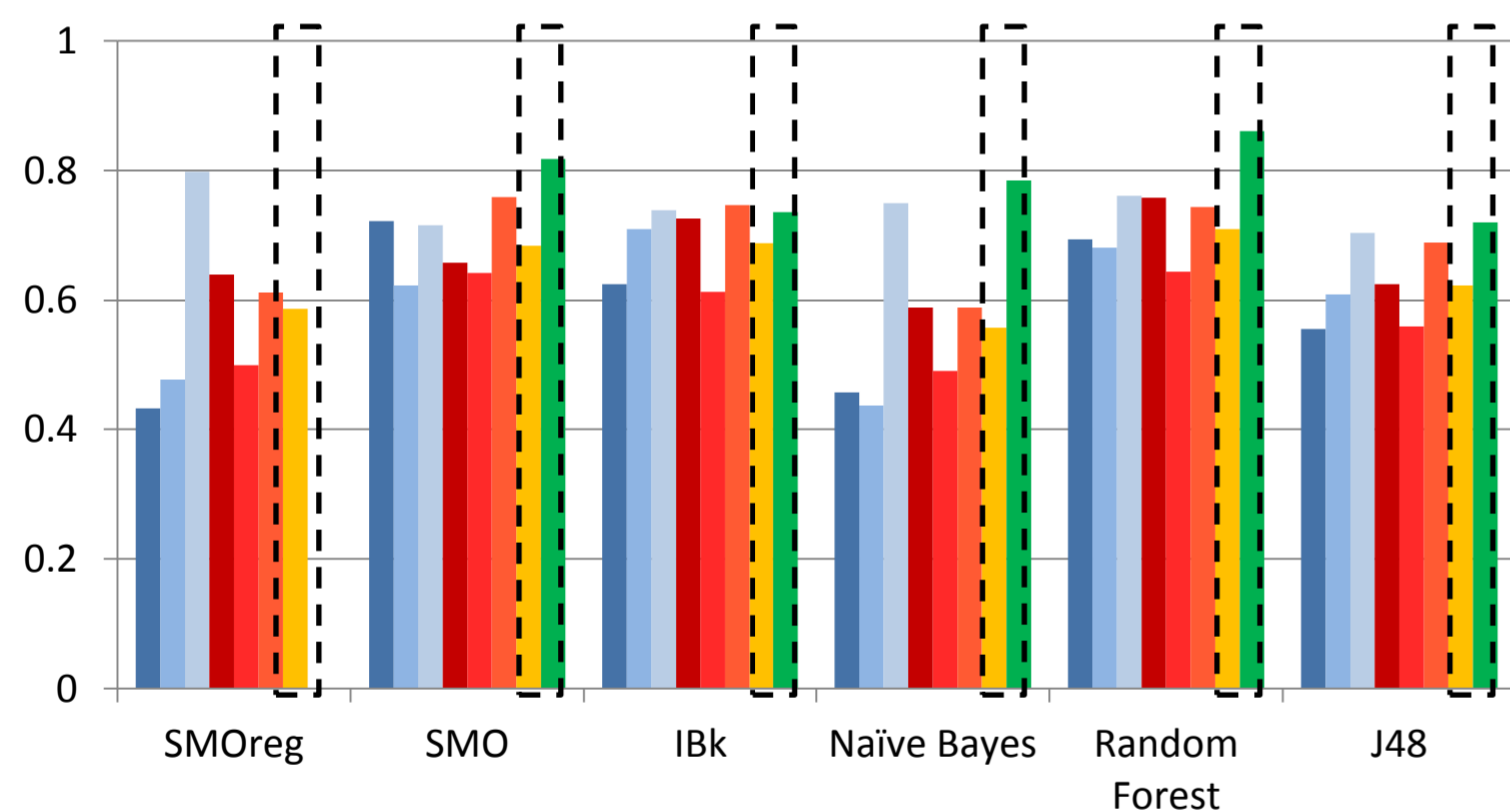

**ExtFP rat**

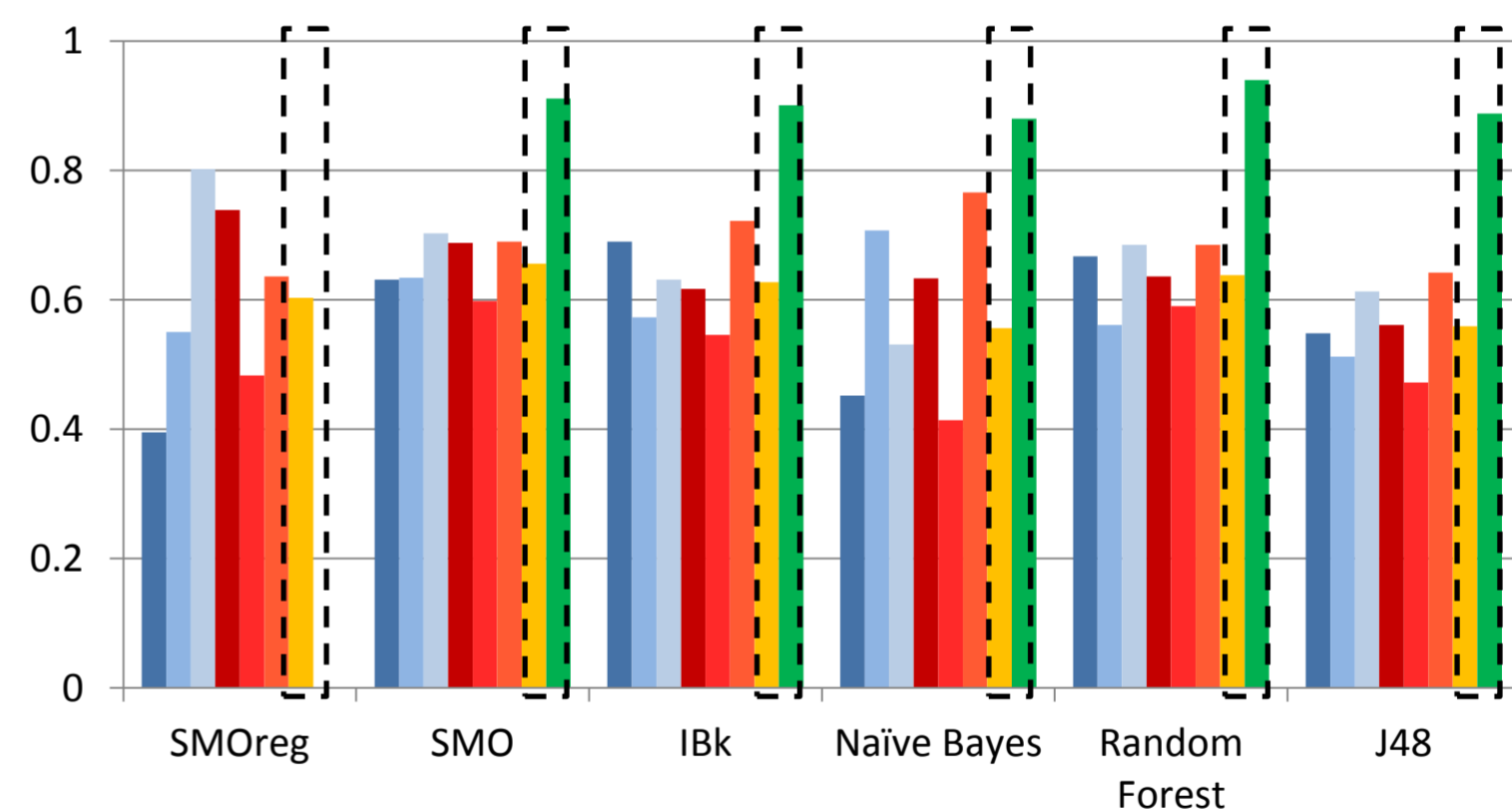

**1d2d mouse**

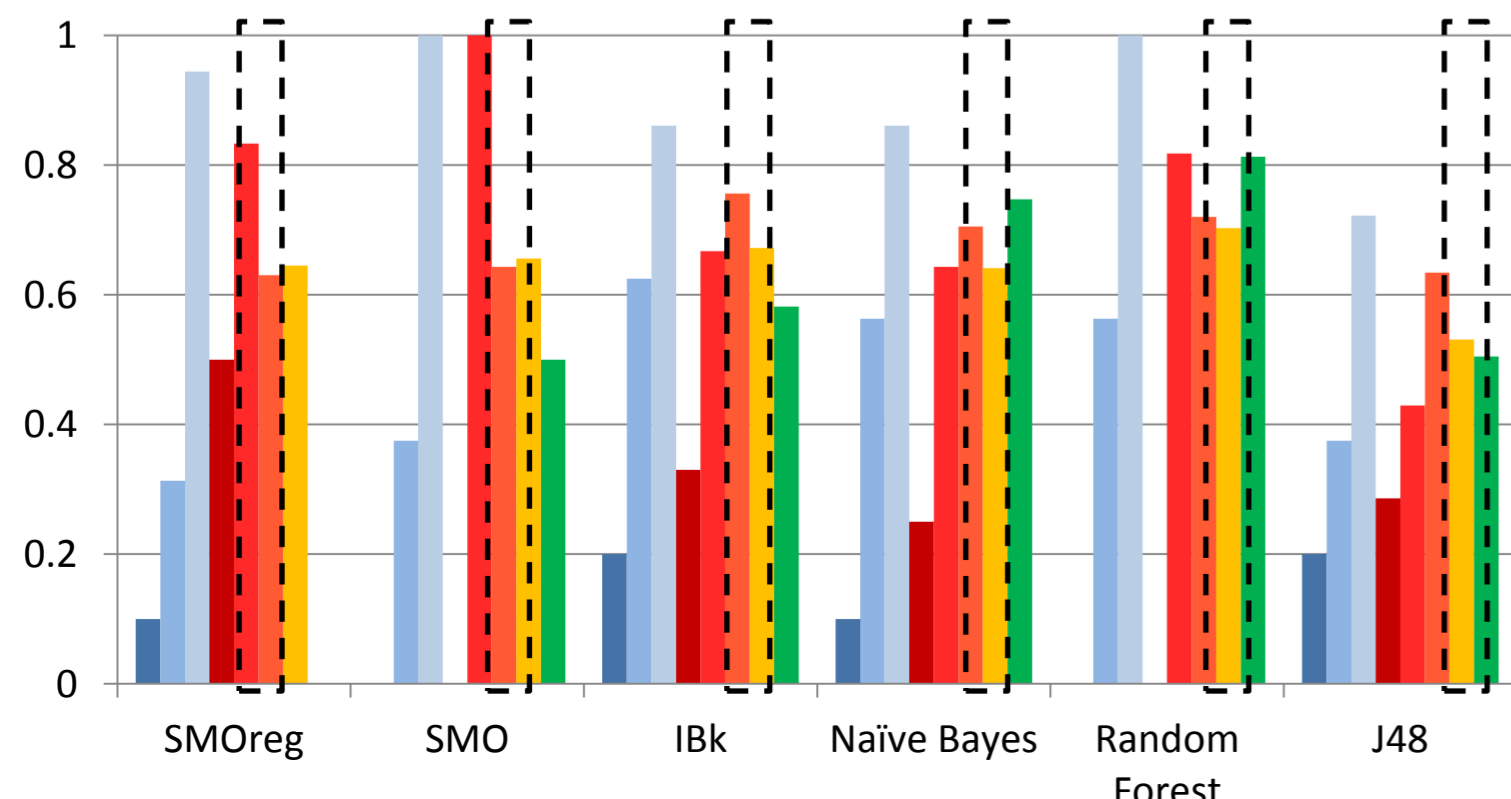

**ExtFP mouse**

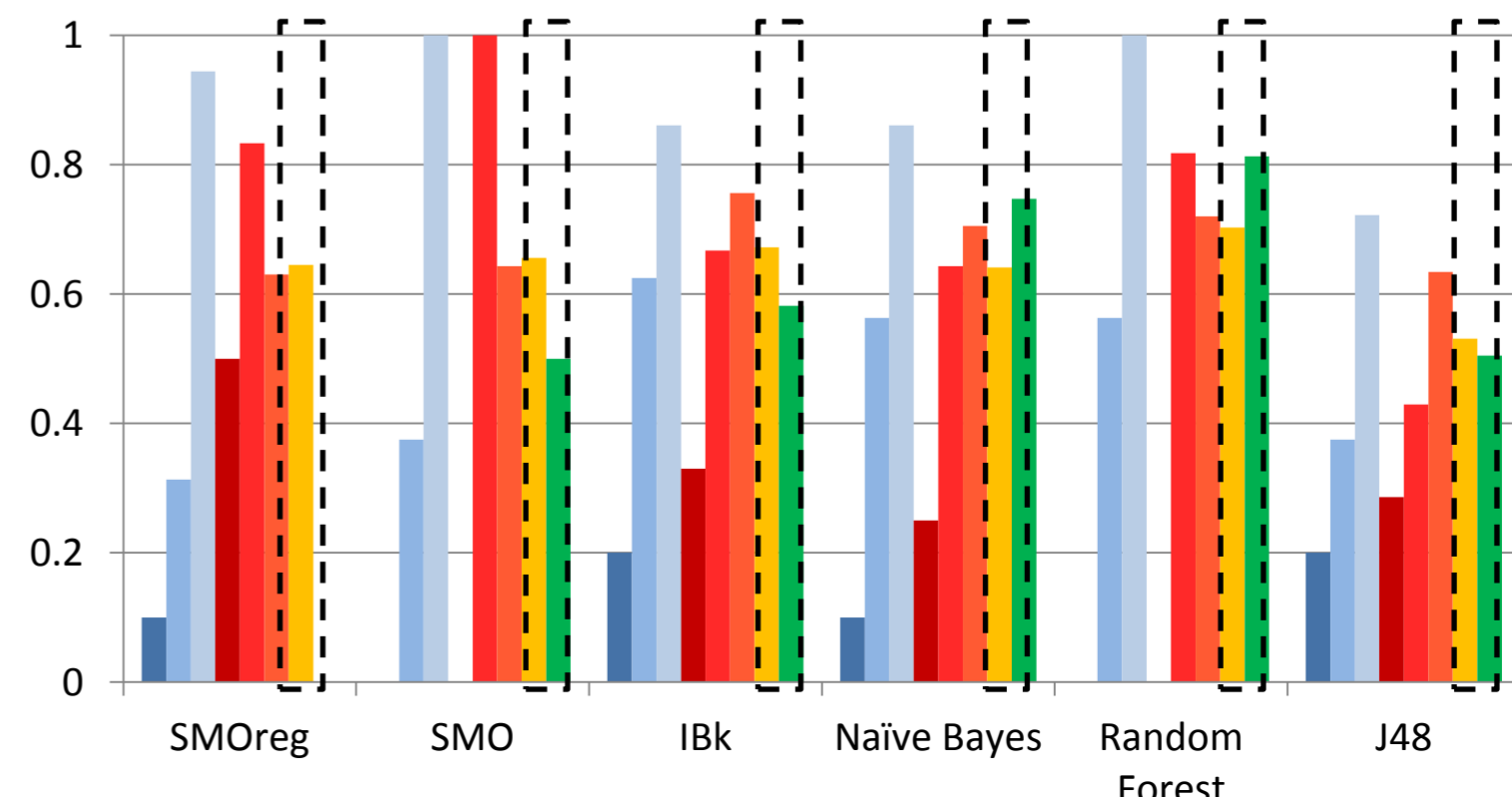

Supplement: Supplementary file 1 [file ijms-19-01040-s001.zip › Supplementary_Material/File_S13_Pdf_file.pdf]

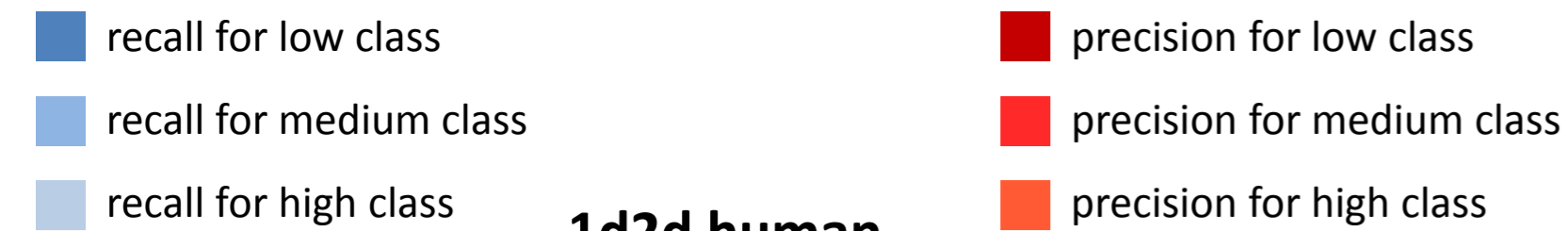

1d2d human

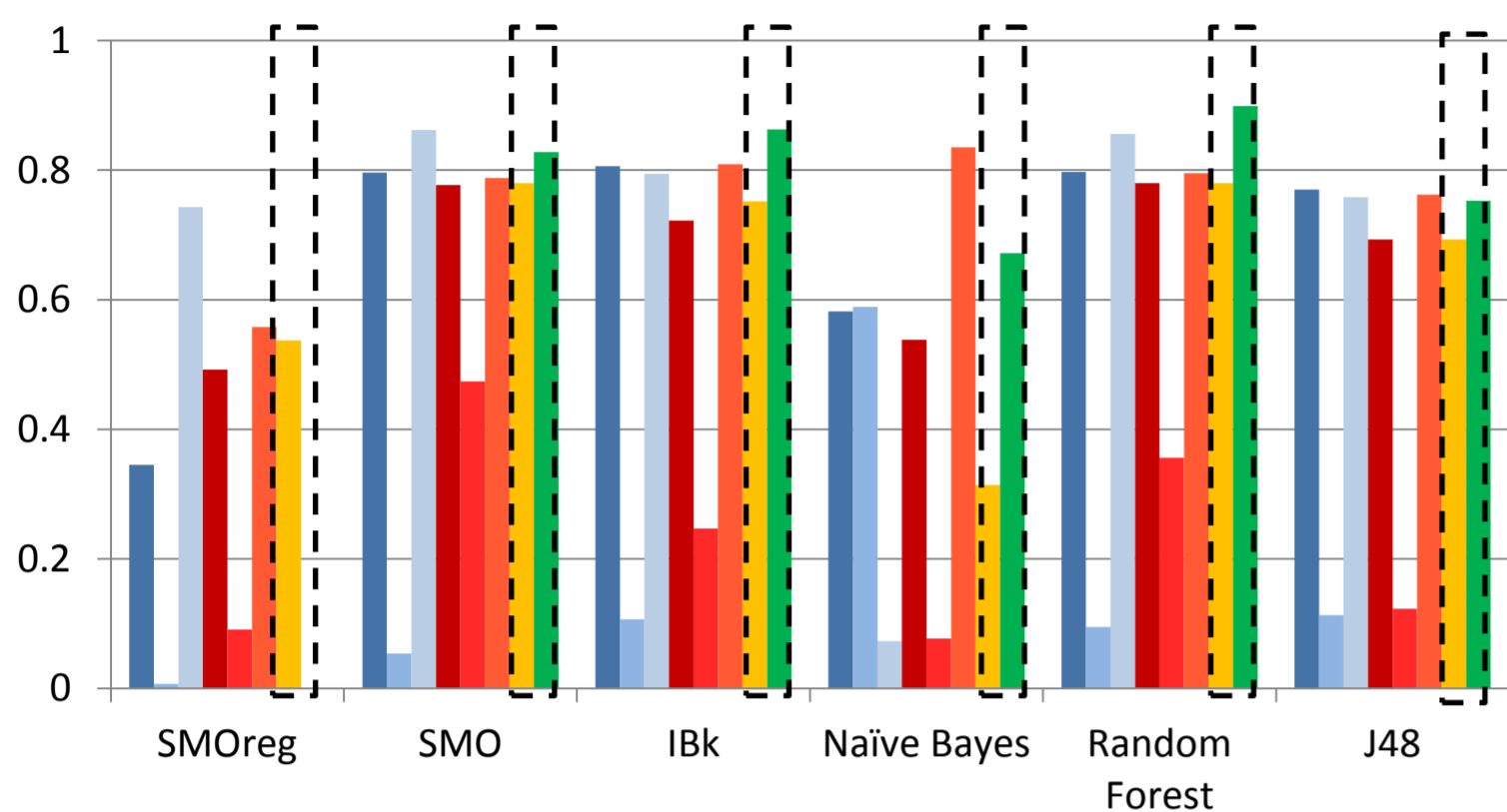

1d2d rat

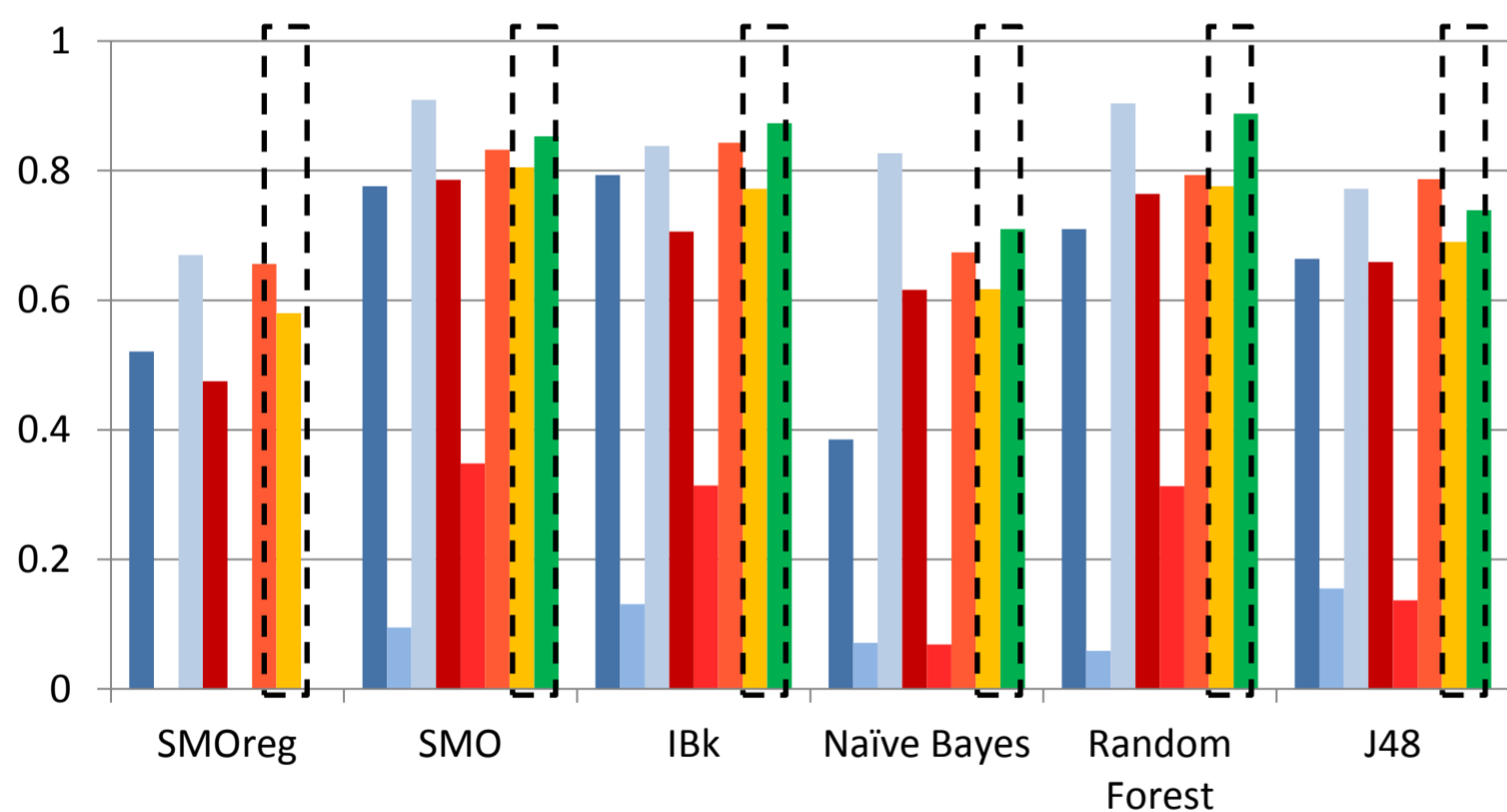

1d2d mouse

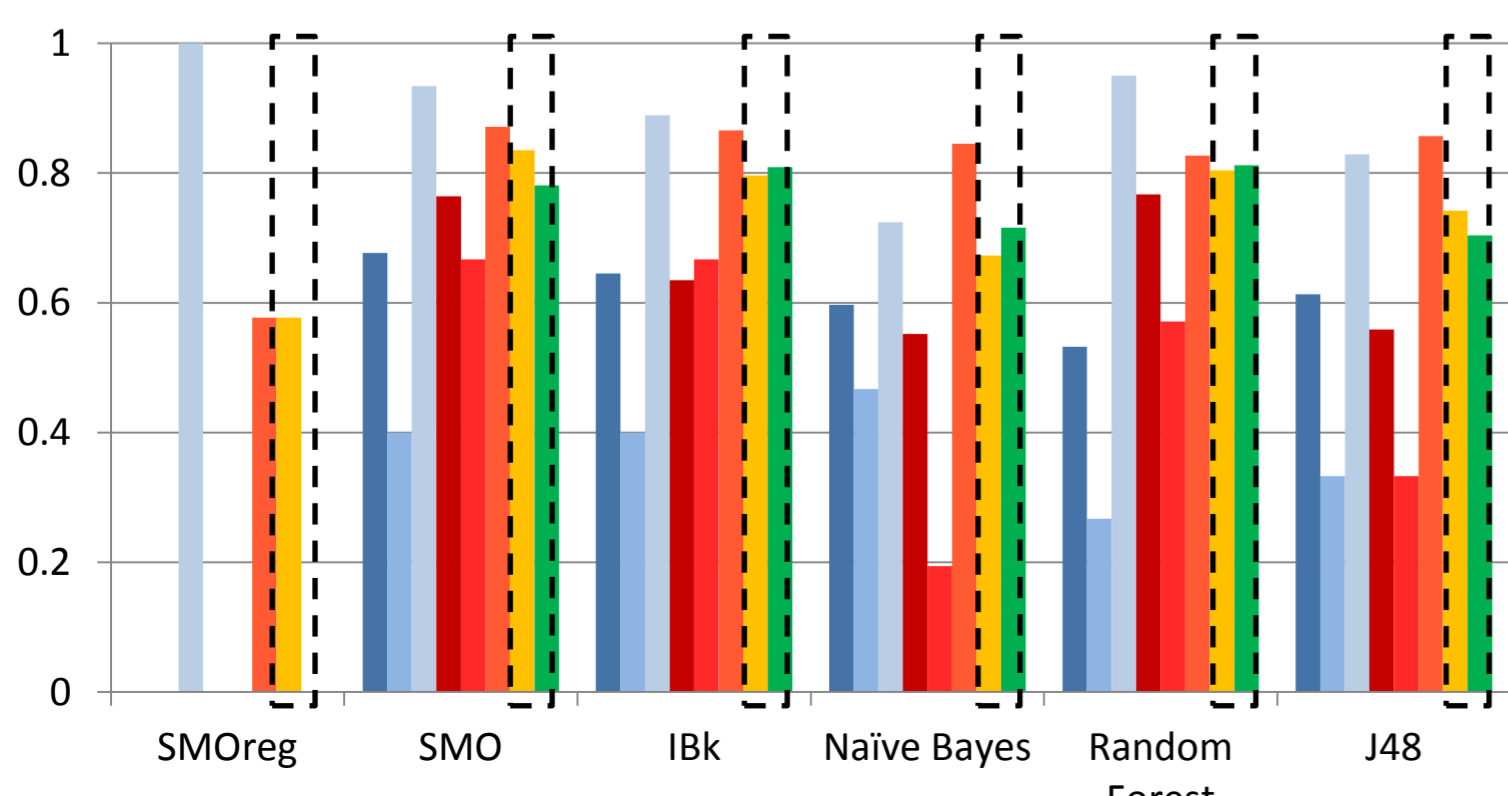

overall accuracy (yellow)

AUROC (green)

ExtFP human

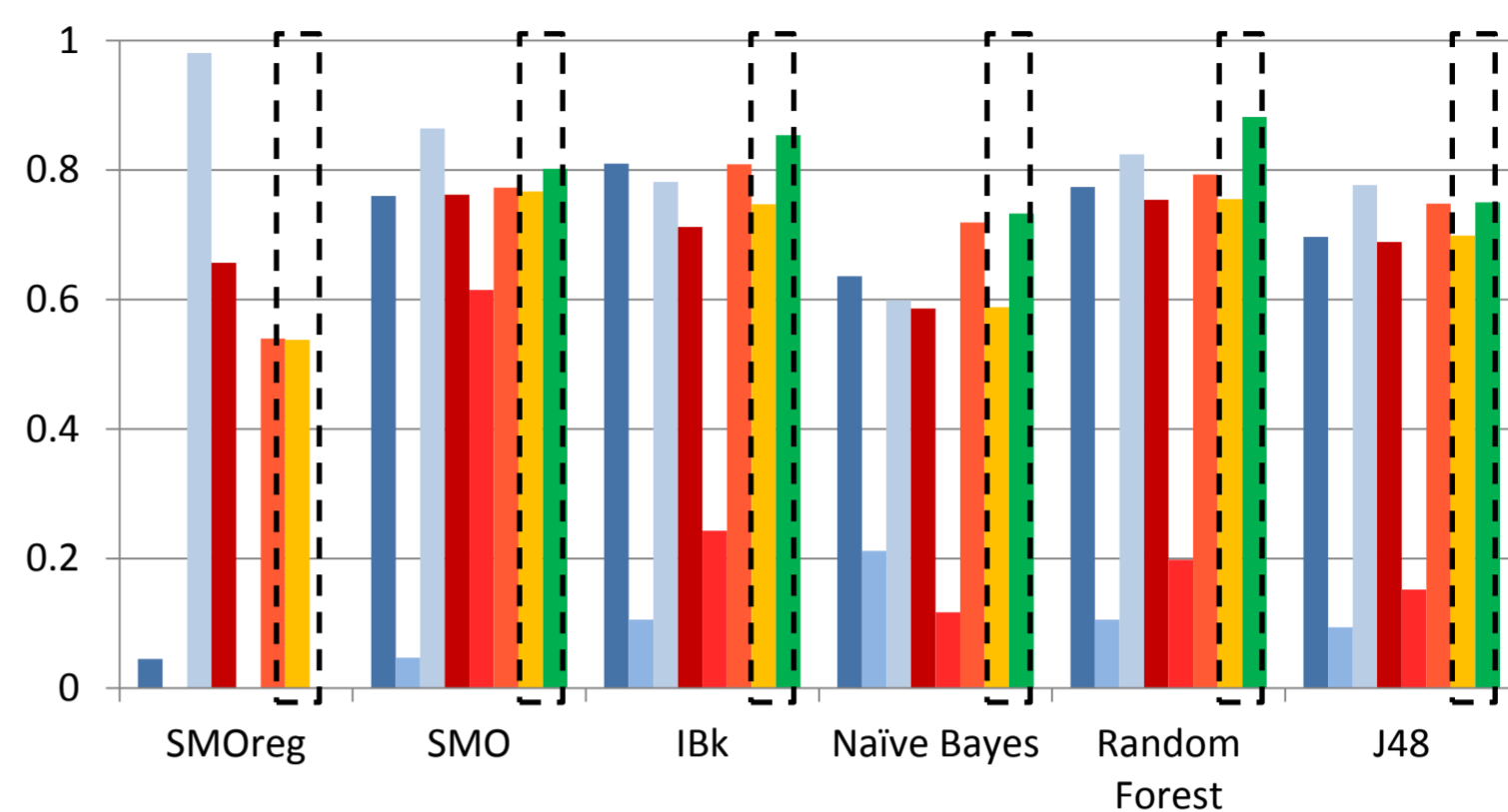

ExtFP rat

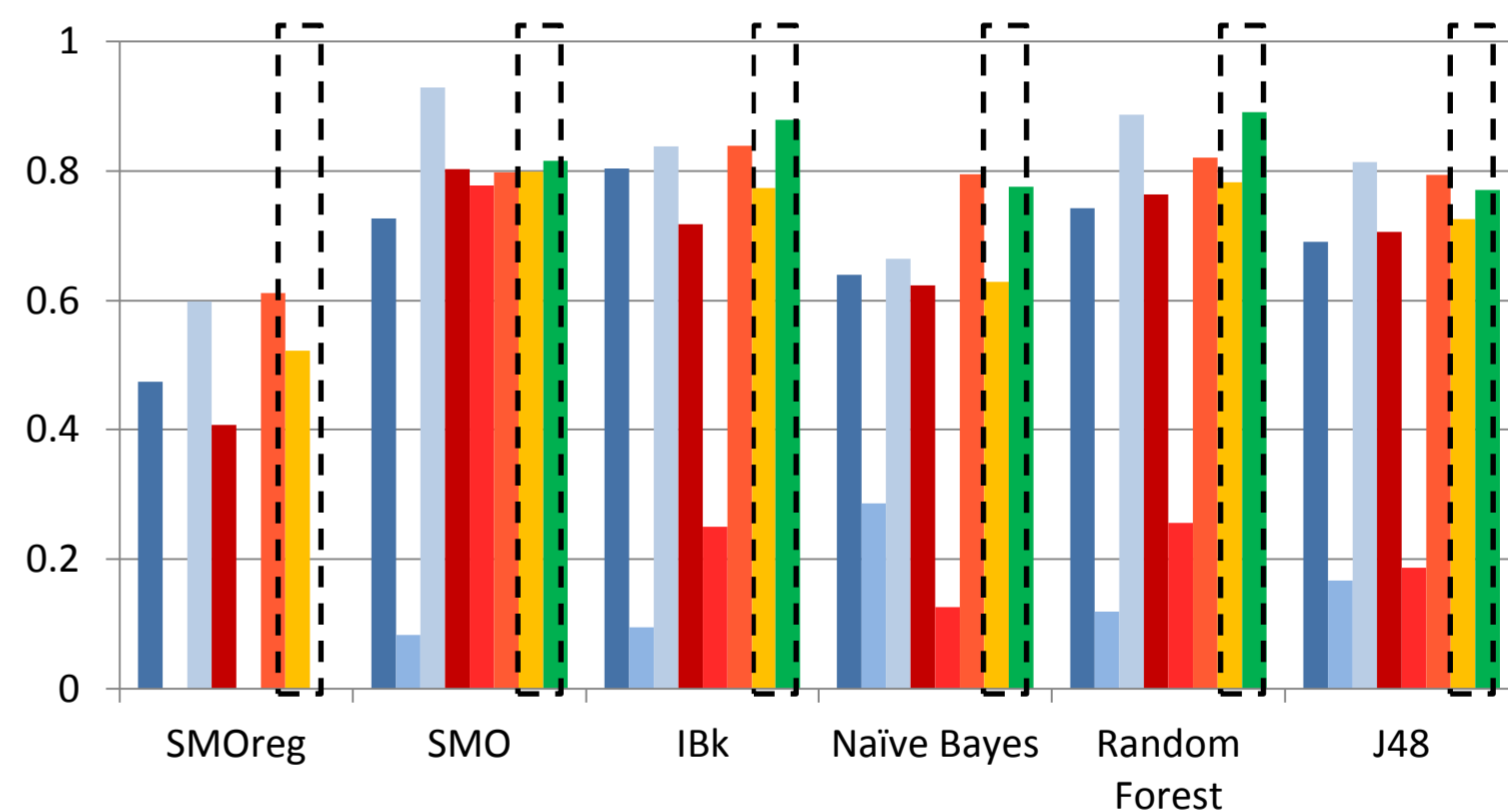

ExtFP mouse

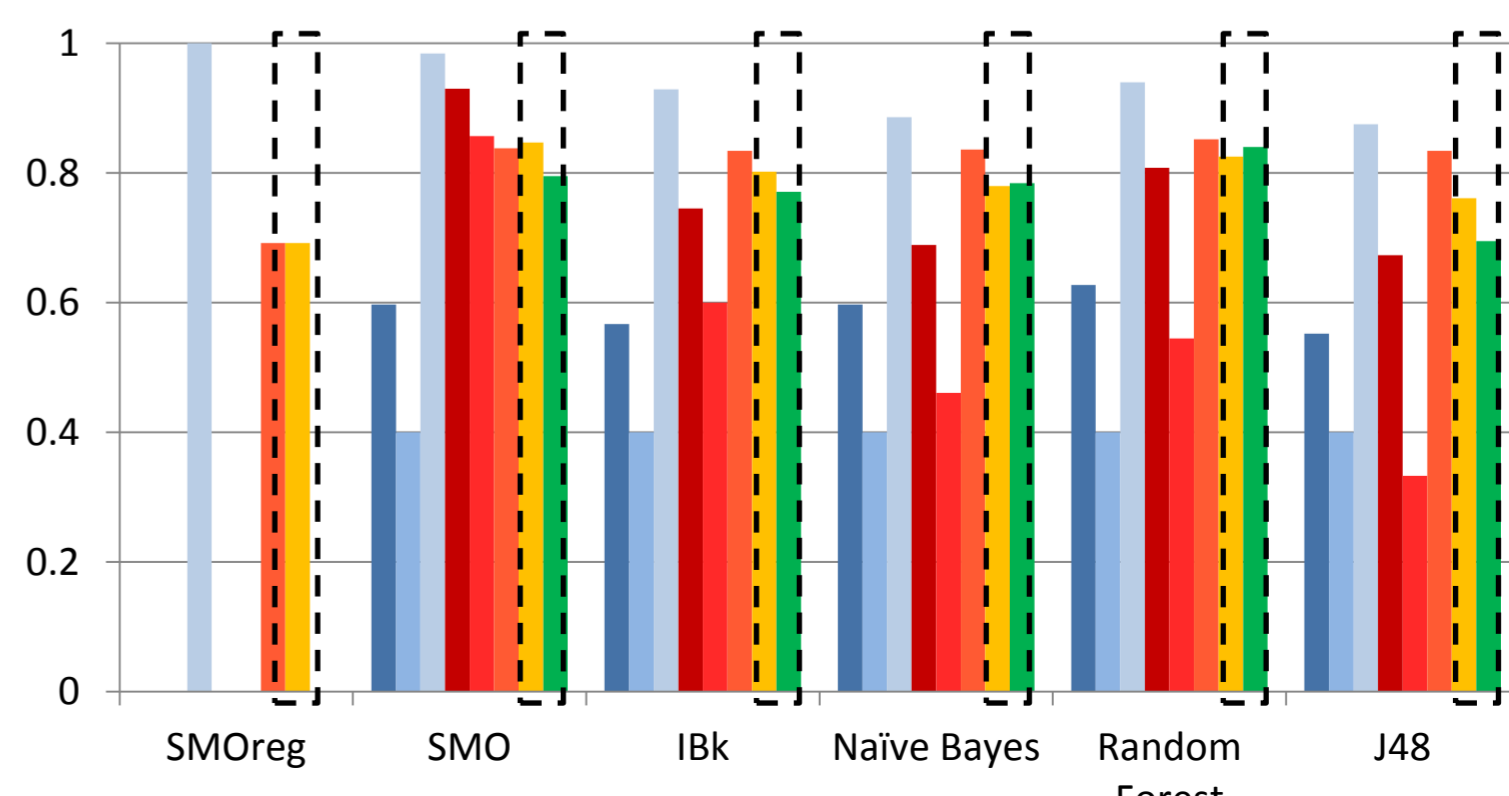

Supplement: Supplementary file 1 [file ijms-19-01040-s001.zip › Supplementary_Material/File_S14_Pdf_file.pdf]
